# Malaria and respiratory syncytial virus as causes of acute febrile illness in an urban paediatric population in Ghana

**DOI:** 10.5281/zenodo.10878344

**Published:** 2014-02-01

**Authors:** Keziah L. Malm, Kofi M. Nyarko, Ernest Kenu, Constance Bart-Plange, Kojo Koram, J.O. Gyapong, Seth Owusu-Agyei, George Armah, Fred N. Binka

**Affiliations:** 1 National Malaria Control Programme, Ghana health service, Accra, Ghana; 2 Non-communicable disease unit, Ghana health service, Accra, Ghana; 3 Department of Medicine, Korle-Bu teaching hospital, Ministry of Health, Accra, Ghana; 4 Noguchi Memorial Institute for Medical Research, University of Ghana, Accra, Ghana; 5 Research Innovation and Development, University of Ghana, Accra, Ghana; 6 Kintampo Health Research Centre, Ghana health service, Kintampo, Ghana; 7 University of Health and Allied Sciences, Ho, Ghana

## Abstract

**Background:**

The sub-Saharan region of Africa is endemic for malaria, and fever is often assumed to be malaria. In Ghana, about 3.7 million cases were reported in 2011, with 24.4% of these laboratory-confirmed. Other causes of febrile illness, including respiratory syncytial virus (RSV), are prevalent in developing countries like Ghana. There is very little data on the prevalence of this virus in the country. This study determined the proportion of acute febrile illness in an urban paediatric population that was due to malaria or RSV.

**Methods:**

A hospital based surveillance system recruited children below five years of age reporting with fever (axillary temperature ≥ 37.5°C) at the outpatient department of an urban hospital from February 2009 to February 2010. Consenting parents/guardians were interviewed, the medical history of the child was taken and the child clinically examined. Thick blood film from capillary blood taken through a finger prick, was Giemsa-stained and microscopically examined for malaria parasites to confirm malaria diagnosis. Nasopharyngeal aspirate was also examined for RSV by polymerase chain reaction.

**Results:**

Out of 481 febrile children, 51(10.8%) were positive for malaria whilst 75 (15.4%) were positive for RSV. Seven of the 75 RSV-positive cases (9.3%) were co-infected with malaria. Based on judgement by clinicians, over 80% of the febrile children were diagnosed and treated as having malaria either alone or in combination with other diseases.

**Conclusion:**

Not all febrile episodes in malaria-endemic regions are due to malaria. The diagnosis and subsequent treatment of patients based solely on clinical diagnosis leads to an over diagnosis of malaria. Improvement in the guidelines and facilities for the diagnosis of non-malaria febrile illness leads to improved malaria diagnosis. Clinicians should be looking for other causes of fever rather than treating all fevers as malaria.

## 1 Introduction

Fever, also known as pyrexia, is a common medical sign characterised by an elevation of body temperature above the normal range of 36.5–37.5°C (98–100 °F)[1]. The burden of Acute Febrile Illness (AFI) in tropical settings may be high, sometimes accounting for more than 50% of all diseases presented at the outpatient department [2,3]. Febrile illness in children may range from mild to severe and can be life threatening [4]. A range of diseases may pre-sent as an AFI and these may be infectious or non-infectious of origin [5,6]. It was estimated that in 2008, 41% of deaths in children below five years of age was due to malaria, diarrhoea, and lower respiratory infections [7]. All these diseases usually present with a fever as a common and early symptom.

Over 90% of cases and deaths in sub-Saharan Africa due to malaria occur in children below five years of age and pregnant women [8]. Unfortunately, most of these cases are diagnosed on the basis of clinical symptoms without laboratory confirmation [8]. In Ghana, malaria is the single most important cause of outpatient attendance in health facilities. It was estimated that about 3.7 million cases of malaria occurred in 2011, accounting for 37.7% of all outpatient attendance [9]. In that same year, malaria admissions accounted for 35.8% of all admissions. Only 24.4% of diagnosed cases were tested either by microscopy or rapid diagnostic tests [9]. Cost of treatment for malaria topped the disease-specific expenditure list of the Ghana Health Insurance Authority in 2008, accounting for 21.4% of all payments made by the authority [10]. The practice of diagnosing most febrile illnesses as malaria based solely on clinical symptoms without an appropriate supportive diagnosis has been the norm. The outcome is that the number of malaria cases reported in the outpatient department over the years has remained more or less the same despite the significant scale up of control interventions in the country that may plausibly have led to a decline in transmission intensity similar to what has been observed elsewhere in Africa.

Acute febrile illness is the most common presentation of disease in young children (<5 yrs) in Ghanaian hospitals and is caused by infections by parasites, bacteria, viruses or fungi. Due to the fact that Ghana is endemic for malaria, most febrile illness are commonly assumed to be malaria to the neglect of other possible causes including bacterial or viral infections. There is a dire need to improve the management of AFI in children by improving the detection of aetiological agents of childhood febrile diseases other than malaria.

The respiratory syncytial virus (RSV) has been shown to account for a significant proportion of acute respiratory tract infections in other developing countries [11-13]. In Ghana there is no published data on what proportion of AFI is caused by RSV. Malaria and RSV can both present as a febrile illness with other nonspecific symptoms. The clinical overlap between malaria and pneumonia due to RSV has important implications for case management strategies and evaluation of disease-specific interventions in regions like ours in which both pneumonia and malaria are prevalent.

A few studies have focused on the relationship between malaria and RSV and suggest the absence of an association between malaria and RSV [12,14,15]. These studies, however, are too few to be conclusive. The rationale for this study was to document the role that malaria laboratory testing could play in monitoring malaria control in Ghana with a view to determining the proportions of AFI that were due to malaria or RSV infection in an urban paediatric setting.

## 2 Materials and Methods

A hospital based surveillance system was set up to recruit eligible children (<5 yrs of age) reporting with fever (axillary temperature ≥ 37.5°C at presentation) at the out-patient department of the La hospital between February 2009 and February 2010. Carers of the children reporting to the health facility were consented for enrolment of their children. The consent process involved explaining the study to the caretakers and seeking their willingness to participate in the study. Parents/guardians that refused to participate were offered standard medical care similar to those that participated. Ethical approval was sought from the Ghana Health Service Ethical Review Committee because the children were recruited from a hospital as well as the Ethical Review Board of the Noguchi Memorial Institute for Medical Research (NMIMR) where the laboratory tests were undertaken.

Where the carer gave consent, an approved study questionnaire was administered to the carer to elicit basic socio -demographic information. Following administration of the questionnaire the medical history of the febrile child was taken and the physician–on-duty then completed the examination section of the form after examining the child. A capillary blood sample was then taken from a finger prick. For each child, thick films were prepared on slides that were labelled on the frosted end with a unique study identity. The films were air dried and later examined. In addition, a nasopharyngeal aspirate was taken into a mucus trap containing 2 ml of buffered saline solution. This was transported on ice to the laboratory and stored for subsequent testing.

Slides were transported to Noguchi Memorial Institute of Medical Research (NMIMR) in boxes and subsequently stained. The slides were placed in a staining trough and a 3% Giemsa solution in buffered distilled water of pH 7.2 was used to fill the trough to cover the slides for 30-45 min. Clean water was then poured gently into the trough to float the iridescent scum off the surface of the stain. After-wards, clean water was used to rinse thoroughly. Slides were removed from the trough and placed in a slide rack with the film side down to drain and to ensure that the film did not touch the rack [16]. After 15-20 min of drying, the slide was read with a magnification of 1000 under oil emersion. Trophozoites were examined and the number of trophozoites seen in 100 fields was counted against 200 white blood cells and recorded [17]. Three different microscopists validated the microscopic examination.

Respiratory Syncytial Virus detection was done by Polymerase Chain Reaction (PCR). The processes included extraction of RSV genomic material, reverse transcription of RSV single-stranded (ss)RNA into complimentary DNA(cDNA), Amplification of the RSV cDNA, RSV genotyping and agarose gel electrophoresis.

## 3 Results

Blood samples of 481 febrile children were microscopically tested for malaria. There were slightly more febrile boys (54.3%) than girls and the median age was 16 months (interquartile range was 22 months), with the majority of children younger than 12 months of age (36.8%). The majority (63.0%) of the mothers of the children had completed primary education whilst only 5.8% had completed education up to tertiary level; 11.4% had no formal education. Of the fathers, 44.7% had primary education and 6.0% had completed education up to tertiary level.

Of the enrolled children, 12.9% (62/469) were brought to the hospital within 24 hrs of developing fever. 55.1% (265/469) reported to the health facility within 2-3 days, and another 26.4% presented within 4-7days after the fever started (Table 1). The median duration of fever before presenting to the hospital was three days. There were missing responses by 2.5% (12/481) of respondents to this question.

**Table 1.** Time taken before presenting the febrile child at the hospital

Number of Days	n	%
Within 24 hrs	62	12.9
>1 day up to 3 days	265	55.1
>3 days up to 7 days	127	26.4
>7 days	15	3.1
No response	12	2.5
Total	481	100

It was also realised that the majority of children had taken some sort of medication before being brought to the hospital, the commonest being an anti-pyretic, which was given to 80% (385/481) of the children, either alone or in combination with other drugs such as antibiotics, antimalarials and/or cough syrups, as shown in Table 2. Only 6 carers (1.2%) admitted to giving the child herbal medication prior to coming to the hospital. About 48% (234/481) of the children had taken an antipyretic only before reporting to the hospital, whilst 1% (7/481) had taken an antimalarial only. About 33% (159/481) had taken two or more medications, which mostly included antipyretics plus other medications. Only 11.7% (56/481) had not taken any medication before reporting at the hospital.

**Table 2.** Medication taken by children before admission

Medication	% (n)
Antibiotic only	1.7 (8)
Anti-malarial only	1.5 (7)
Antipyretic only	48.6 (234)
Cough mixture only	2.7 (13)
Two or three medications	33.1 (159)
Four different medications	0.8 (4)
No medication	11.6 (56)
Total	100 (481)

Multiple symptom presentation was reported among the cases. Besides fever, the most common symptom presented was nasal discharge, seen in 67.9% (326/481) of cases, whilst the least common symptom was difficulty in breathing among 53.3% (256/481) of the cases. Eighteen percent (86/481) of the children had been given some form of antimalarial either correctly or incorrectly before coming to the hospital. There were significantly more people with negative microscopy who had taken some form of antimalarial compared to those who had not (X2= 9.114, p=0.012).

**Figure 1.**
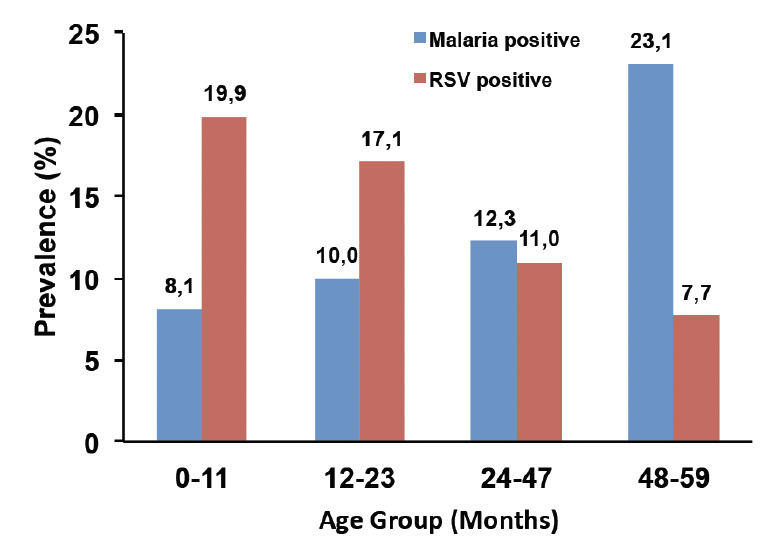
Prevalence (%) of RSV and malaria by age group.

Out of the 481 children whose blood sample was taken for microscopy, 51 were positive for malaria, giving an overall positivity rate of 10.8%. Malaria was equally distributed among boys and girls. Overall, the proportion of malaria cases increased with age and children aged 48-59 months were mostly affected (19%) by malaria with those 0-11months (8.1%) least affected as shown in Fig. 1. Based on clinical judgement, over 80% of these febrile children were diagnosed and treated as malaria either alone or in combination with other diseases by clinicians.

With respect to RSV, 75 children (15.6%) tested positive. The youngest children (less than 12 months) were mostly affected (19.8%, 35/481), as shown in Fig. 1. There were slightly more boys than girls that were positive for RSV but this difference was not significant (p =0.748). The majority (89.3 %) of RSV-positive cases were of the B genotype, 6 (8.0%) were of genotype A and 2 (2.6%) were of mixed infection AB. Among the RSV-positive cases, all the children with severe illness were of the genotype B.

**Figure 2.**
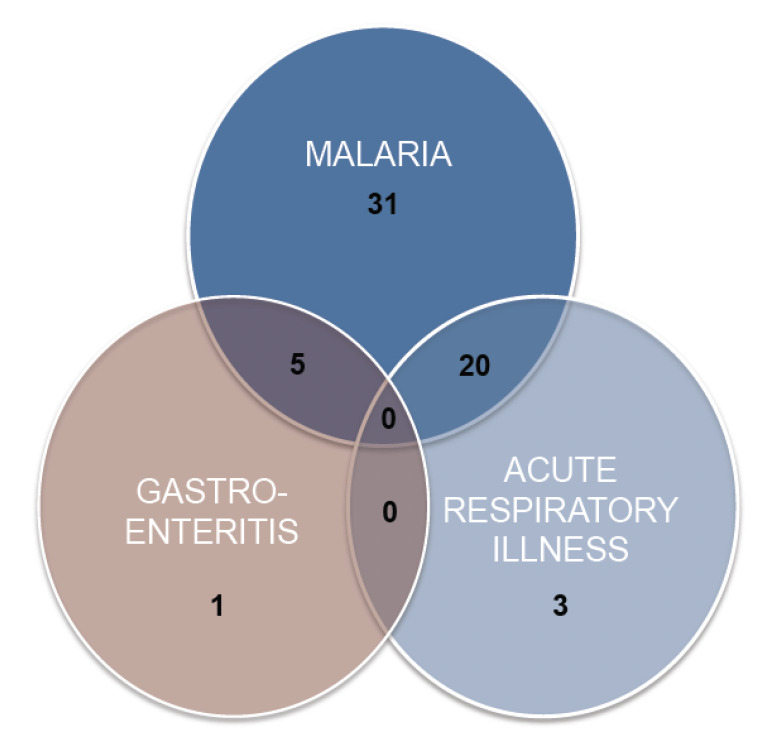
Clinical diagnosis of RSV-positive children made by clinicians during the study period (February 2009 to February 2010).

Seven of the 75 RSV positive cases (9.3%) were coinfected with malaria and all the co-morbid cases were of type B genotype. Five of the co-morbid cases were females and two were male. Two of the cases were less than 12 months and five were aged 12-24 months. None of these children presented with severe symptoms and were all treated on outpatient basis. Fifty-six of the 75 RSV cases (74.6%), however, were diagnosed by clinicians either as malaria or malaria in combination with gastroenteritis or acute respiratory illness (ARI), with only 3 as purely ARI (Fig. 2).

## 4 Discussion

Fifteen percent of the children with febrile illness were found to have RSV infection. Nevertheless, the majority of these cases was diagnosed by clinicians as malaria. This finding compares with Taylor’s [18] assertion that there is a high overlap of the clinical symptoms of malaria and respiratory tract infections.

Based mainly on clinical judgement, 80% of febrile children in this study were diagnosed and treated as malaria either alone or in combination with other diseases. However, based on clinical suspicion confirmed by microscopy, the proportion of febrile illness due to malaria was found to be 10.8%. Treatment based mainly on clinical diagnosis only leads to gross abuse of antimalarials as has been documented in other parts of Africa, including Mozambique and Tanzania [19-21]. Clinicians generally treat patients based on presumptive diagnosis mainly due to peer-influence and pressure as well as expectations from patients [21]. There is enough evidence, based on published studies, that when diagnosis of febrile illness is based on clinical symptoms only, more than 50% of cases treated as malaria may not be malaria [22,23]. Such practices have led to outpatient department (OPD) malaria cases always being high;; the Ghanaian National Malaria Control Programme report for 2011 documented as high as 37.7% of all OPD classified as malaria because most diagnoses are presumptive [9]. The treatment of febrile cases based solely on clinical symptoms has been shown to be less cost-effective compared with confirmed laboratory diagnosis [24];; it also promotes the development of drug resistance [25]. Even in some cases, where laboratory results are negative, clinicians have still gone ahead to prescribe antimalarials because of the assertion that malaria is much more easier and acceptable to diagnose than other illnesses and it is seen as unpardonable to miss a case of malaria [21,25]. This practice could explain the high proportion of funds that the Ghana National Health Insurance Authority (NHIA) spends on the treatment of malaria alone [10].

Our finding that the proportion of fever due to malaria is low compared to what was detected in other studies indicates clearly that not all fever is malaria, even in the endemic regions [26-28]. Even if those who took antimalarials prior to coming to the hospital in this study were considered as false negatives, the proportion of febrile illness due to malaria was still low [29]. Presumptive diagnosis could be associated with improper management of fevers that may have fatal consequences [22,27]. Our study, together with the findings of other studies [24,26,27], emphasises the need for confirmation of malaria diagnosis by a laboratory test. In order for clinicians to be able to do this, however, there is the need for an effective, easy to use and quick laboratory test. In a resource-limited country where microscopy may not be available in all areas, the use of rapid diagnostic tests can improve diagnosis [28-30].

Evidence from Senegal shows that massive scale up of RDT usage resulted in a reduction in ACT consumption because clinicians generally followed guidelines [31]. It is therefore important that clinicians adhere to guidelines for diagnosis and management of malaria febrile illness but also that the tools for the diagnosis of non-malaria febrile illnesses improve. Efforts should be made by the global scientific community to develop and make available rapid diagnostic tests for identifying non-malaria febrile diseases. This will help greatly in improving the care of malaria febrile illness. Malaria-RSV co-infection was low in this study and this is similar to what was found in Mozambique and Kenya [12,14]. This group of children did not present with more severe symptoms than those affected by only RSV or malaria alone. There is the possibility that RSV infection suppresses malaria but more work needs to be done to confirm this.

## 5 Conclusions

Clinicians treated the majority of children who presented with fever at a Ghanaian hospital as malaria. The perception that almost every fever is malaria in malaria-endemic countries should be dismissed. This study showed that most febrile illnesses in an urban paediatric population are not due to malaria and highlights the need for improvement in diagnosis of non-malaria causes of febrile illness and the adherence to guidelines for such febrile illness by clinicians. Clinicians should be looking out for other causes of fever, like RSV.
